# Decoding Vagus-Nerve Activity with Carbon Nanotube Sensors in Freely Moving Rodents

**DOI:** 10.3390/bios12020114

**Published:** 2022-02-11

**Authors:** Joseph T. Marmerstein, Grant A. McCallum, Dominique M. Durand

**Affiliations:** Neural Engineering Center, Biomedical Engineering, Case Western Reserve University, Cleveland, OH 44106, USA; jtm124@case.edu (J.T.M.); gam19@case.edu (G.A.M.)

**Keywords:** vagus nerve, intraneural, decoding, intrafascicular, recording, carbon nanotube

## Abstract

The vagus nerve is the largest autonomic nerve and a major target of stimulation therapies for a wide variety of chronic diseases. However, chronic recording from the vagus nerve has been limited, leading to significant gaps in our understanding of vagus nerve function and therapeutic mechanisms. In this study, we use a carbon nanotube yarn (CNTY) biosensor to chronically record from the vagus nerves of freely moving rats for over 40 continuous hours. Vagal activity was analyzed using a variety of techniques, such as spike sorting, spike-firing rates, and interspike intervals. Many spike-cluster-firing rates were found to correlate with food intake, and the neural-firing rates were used to classify eating and other behaviors. To our knowledge, this is the first chronic recording and decoding of activity in the vagus nerve of freely moving animals enabled by the axon-like properties of the CNTY biosensor in both size and flexibility and provides an important step forward in our ability to understand spontaneous vagus-nerve function.

## 1. Introduction

The vagus nerve innervates nearly every internal organ, providing sensory input to the brain and parasympathetic-control inputs to the viscera. Therefore, abnormal vagus-nerve activity has been linked to many chronic diseases, such as epilepsy, diabetes, hypertension, and cancer [1,2,3,4,5]. Vagus-nerve stimulation has been used to treat a wide variety of diseases [6], most successfully implemented for the treatment of epilepsy [7], even while the mechanisms are not well understood and direct recordings of vagal activity associated with disease are not available [8]. The majority of vagal afferent fibers come from the gut [9,10], and abnormal vagal activity has been clearly implicated in eating and metabolic disorders [11,12,13,14,15]. In this study, we analyze the first chronic recordings of vagal spikes and the correlation of signals to several behaviors in healthy rats.

The chronic recording of vagal signals has been limited, partially due to the difficulty in chronically recording high-quality signals in small autonomic nerves. Extraneural cuff electrodes have proven to be very effective peripheral nerve interfaces, allowing for selective stimulation [16] and some selectivity in recording [17,18,19,20]. However, the insulating perineurium layer between the active nerve fibers and the recording electrodes results in a low signal-to-noise ratio (SNR) or requires desheathing of the nerve. Thus, intraneural or intrafascicular electrodes placed much closer to active fibers may be necessary for certain recording applications, providing a higher SNR and higher selectivity for multifascicular nerves. However, intraneural electrodes are more invasive and thus, have issues related to long-term stability [21,22,23,24,25]. In particular, small autonomic nerves necessitate smaller and more flexible neural sensors. We have previously shown that carbon nanotube yarn (CNTY) electrodes have favorable properties for nerve interfacing: specifically, their small size, high flexibility, and low impedance. Thus, they provide a stable, high-SNR interface for chronic recording in small autonomic nerves in rats, with high-quality signals continuing up to four months after implantation [26]. Furthermore, we have developed techniques for recording activity in the cervical vagus nerves of rats without anesthesia, allowing for the first chronic recordings of truly spontaneous vagal activity. This technology has been successfully applied to make the first direct measurements of vagal tone in freely moving animals [27].

Due to the prevalence of gastric afferents in the vagus nerve, we expect signaling from the gut to be the dominant activity present in vagal recordings. Previous studies have shown that vagal afferents are sensitive to mechanical stimulation of the gut [28] and to gastric hormones which regulate food intake and gastric motility [29,30,31]. There are also reflex pathways which modulate efferent vagal activity in response to gastric distention and contractions [32]. However, such signals have not been reported from chronic, unanesthetized animals, and this CNTY-electrode biosensor demonstrates the ability to decode vagal activity related to various animal behaviors, such as eating.

In this study, we continuously record spontaneous vagal-spiking activity from awake, freely moving rats for >48 h up to two weeks after implantation. To our knowledge, this is the first time this has been successfully demonstrated. The neural-recording data was synchronized with continuous video recording of the subjects. Spike sorting is used to separate semi-distinct spike clusters, which are then correlated to animal behavior identified from the video recordings. Interspike interval distributions are also found to change in response to food intake, presenting another neural feature that can be used to decode spontaneous vagal activity. We report several spike clusters that show tuning to animal eating, and the firing dynamics of multiple decoded spike clusters can be used to classify eating compared to drinking, grooming, and resting behaviors.

## 2. Materials and Methods

### 2.1. CNTY Electrode Manufacture

**Figure 1:**
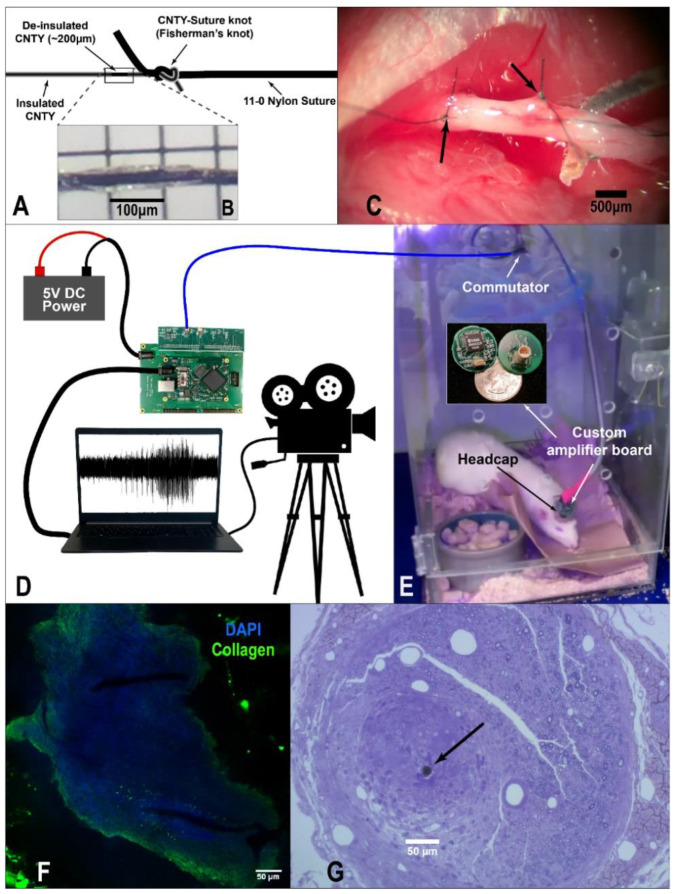
Electrode implantation, histology, and recording methods. (**A**) Diagram of CNTY electrode mated with an 11-0 nylon suture with a fisherman’s knot. (**B**) Section of CNTY electrode deinsulated by laser. (**C**) Vagus nerve with two implanted CNTY electrodes. CNTY-suture knots are shown with arrows. (**D**/**E**) Diagram showing the setup for continuous recording of vagal activity and video for behavior identification. Signals travel from the implants to the headcap connector mounted on the animal’s skull, where they are digitized and amplified by the custom amplifier board shown. These signals are then routed through a commutator, which can rotate and allows the animal to move freely without twisting or pulling on the cable. From the commutator, the signals are sent to an Intan USB interface board, which is powered by an external DC-power source and finally sends the signals to a computer, where they are saved and can be viewed in real time. A video camera is manually synced to the vagal recordings. (**F**) Fluorescent images showing collagen + cellular encapsulation of CNTY electrodes implanted in the vagus nerve for seven days. (**G**) Toluidine blue-stained nerve section showing encapsulation of a CNTY electrode implanted for two weeks.

CNT yarns were manufactured at Case Western Reserve University, as described previously [26]. CNTYs were then connected to 35NLT^®^-DFT^®^ wire (Fort Wayne Metals, Fort Wayne, IN, USA) with silver conductive epoxy (H20E, EPO-TEK), creating a CNTY-DFT^®^ junction. Dacron mesh and silicone elastomer (MED-4211/MED-4011, NuSil Silicone Technology, Carpinteria, CA, USA) were added to seal the junction, confirmed by measuring the impedance of the junction at 1kHz in a saline bath. The free end of the CNTY was tied to the end of an 11-0 nylon suture (S&T 5V33) using a fisherman’s knot, as shown in Figure 1A. The entire CNTY was coated with parylene-C (5 µm thick vapor deposition coating, SMART Microsystems, Elyria, OH, USA) on a custom rack which masks the suture needle from coating. Then, a small section (~200 µm long) of parylene-C was removed approximately 500 µm behind the CNTY-suture knot using a laser spot welder (KelanC Laser, set to 1A current, 0.3 ms pulse width, and 300 µm diameter), as shown in Figure 1B. Figure 1C shows the CNTY-suture knot outside of the nerve after implantation. Electrode viability was confirmed by measuring the impedance of the recording site before and after using the laser.

### 2.2. Surgery

All surgical and experimental procedures were done with the approval and oversight of the Case Western Reserve University Institutional Animal Care and Use Committee to ensure compliance with all federal, state, and local animal welfare laws and regulations. Electrodes were implanted in male Sprague Dawley rats between 7–12 weeks of age.

To expose the left cervical vagus nerve, a midline incision was made along the neck. The muscles and salivary glands were separated and held in place, revealing the carotid sheath which contains the carotid artery and vagus nerve. The vagus nerve was carefully separated from the carotid artery using blunt dissection and held in slight tension using a glass hook. CNTY electrodes were implanted by sewing the suture through the nerve for ~2 mm, then pulling the suture until the CNTY-suture knot was pulled through. Then, the electrode was pulled back so that the knot sat against the epineurium, ensuring the recording site remained inside the nerve, as shown in Figure 1C. Two electrodes were implanted with ~2 mm separation; the extra suture and needles were cut off after implantation, and the nerve, electrodes, and junctions were covered with ~1 mL of fibrin glue (Tisseel, Baxter International Inc., Deerfield, IL, USA) to help secure the area for recovery. Next, the DFT wires were tunneled from the neck to the back of the skull and soldered to a 5-pin Omnectics connector (Omnetics Connector Corporation MCP-5-SS). The skin on top of the skull was opened, and the connector was fixed on top of the skull with dental cement. The amplifier ground was connected to a screw placed in the skull, which also helps keep the headcap in place. Electrodes were implanted for chronic recording in two animals, and animals were given one week for recovery before recording.

### 2.3. Recording

Recordings were carried out continuously in awake, behaving animals for 56 and 40 h (Rat 1 and 2, respectively). A custom-built PCB with an Intan RHD2216 recording chip was attached to the headcap connector, which was secured to the animal with a 3D-printed locking mechanism and attached to a PlasticsOne^®^ (Roanoke, VA, USA) commutator, allowing the rat to move around the cage without tangling or pulling on the connector cable [27]. Input signals were routed to eight amplifier channels, using 8-channel hardware averaging to decrease amplifier noise. Output from the amplifier board was run through the commutator into an Intan RHD USB Interface board (Intan part #C3100), which is powered by an external battery supplying 5V DC power. Signals are then routed to a computer where they are saved for offline analysis and can be viewed in real time.

Neural recordings were sampled at 20 kHz with a 5 kHz low-pass filter. Recordings were started around 10 AM (approximately four hours after the start of the light cycle). During ENG recording, a video camera was used for simultaneous video recording. The camera was equipped with an infrared light and infrared sensor, allowing for filming even during the dark cycle. The camera was connected to the recording computer and manually synced to the recording. A diagram of the recording setup can be seen in Figure 1D,E.

### 2.4. Signal Processing

**Figure 2:**
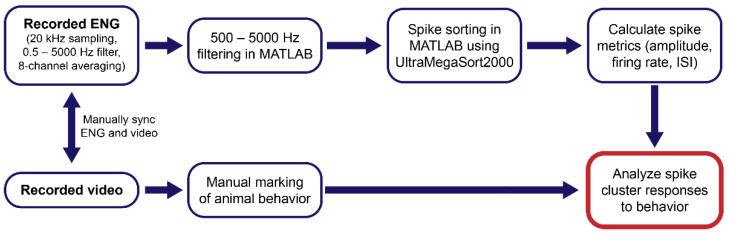
Diagram of data processing and analysis workflow. Vagal ENG and video are recorded simultaneously from freely moving rats. Spike sorting is used to decode spike metrics, which are analyzed with respect to animal behaviors identified from the video.

ENG data were imported into MATLAB, where they were further processed. ENG was band-pass filtered from 500–5000 Hz to minimize interference from EMG, ECG, or other possible sources. The filter bandwidth was kept relatively wide to minimize distortion of spike waveforms. Spikes were detected and sorted into clusters using the UltraMegaSort2000 software in MATLAB, using a threshold of eight times the RMS of baseline. Spike waveforms (3 ms long) were transformed into the principal component space, and principal components accounting for 95% of the total waveform variance were used for spike clustering. Spike clustering was done using k-means clustering of spike waveform principal components, with a maximum of k = 256 clusters. Using the UMS2000 software, clusters were further analyzed for better separation and exclusion of artifacts. First, outliers were removed if they had a z-score greater than 500 on the χ^2^ distribution of distance to the cluster center. Clusters were removed from analysis if the spike waveform contained a second, larger threshold crossing (i.e., removing of spikes which were detected twice due to threshold-crossing of the spike tail). Clusters were also removed if spike width was less than 0.3 ms and amplitude was greater than 1mV (presumed recording artifacts) or if spike width was greater than 2 ms. Spike waveform values were used to calculate spike amplitudes (difference between the maximum and minimum voltage values) and the spike RMS. Spike-cluster-firing timings were also used to calculate cluster-firing rates and interspike intervals (ISI). Average spike amplitudes over time are shown in Supplementary Figure S1, and spike RMS was used to calculate average SNR, shown in Supplementary Figure S2. Animal behaviors (eating, drinking, grooming, and resting) were identified via video recording. The overall data processing and analysis workflow is diagrammed in Figure 2.

### 2.5. Histology

Toluidine blue staining: the image shown in Figure 1G was obtained from an implanted nerve which was fixed, sectioned, and stained with toluidine blue. Two weeks after implantation, animals were perfused with 1.25% glutaraldehyde, 1% formalin, and 0.1 M phosphate buffer. This fixative solution is approximately 640 mOsM/kg. Animals were injected with 0.2–0.5 mL of 1% procaine at 37 °C through the left ventricle. Followed by 200 mL of the fixative solution perfused at 37 °C using a variable speed peristaltic pump. After completing the perfusion process, the vagus nerve was dissected at the implant location. The complete nerve section was transferred into a postfixative solution (1% osmium tetroxide in 100-mM phosphate buffer) for two hours at room temperature before being transferred to 4 °C. Following postfixation, the nerve tissue was dissected in 1-mm-long pieces and embedded in an epoxy resin. Sections (0.7 m) were cut from the epoxy blocks using a diamond knife (DiATOM) microtome. Toluidine blue (1% toluidine blue and 2% borate) was used to stain the nerve axons.

Fluorescent staining: the image shown in Figure 1F was obtained from an implanted nerve which was optically cleared using the CLARITY protocol [33,34]. Seven days after implantation, the vagus nerve was extracted and immediately placed into hydrogel monomer solution. The sample was passively cleared and stained with a collagen antibody, as described by our group previously [26]. DAPI staining was done by placing the sample in VectaShield with DAPI (Vector Laboratories) on a glass-bottom petri dish (Ted Pella, Inc., Redding, CA, USA). Samples were imaged on a Leica SP8 gSTED Super-Resolution Confocal microscope (Leica Microsystems, Wetzlar, Germany).

### 2.6. Statistical Methods

Where relevant, results are reported as mean ± standard deviation. Average spike waveforms in Figure 2 are shown with shaded areas representing the 95% confidence interval. Overall spike-firing rate, median spike amplitudes, and average spike SNR over time were fitted with a linear regression to determine if the slope was different from zero, with slopes and *p*-values shown in Figure 2 and Supplemental Figures S1 and S2. Spike clusters were grouped based on their response to eating, and firing rate changes before, during, or after eating for each group were compared to baseline group firing rates using a one-sample *t*-test, with a significance level of 0.01 and a Bonferroni correction (α = 5.6 × 10^−4^), as shown in Table 1. ISI distributions of the before, during, and after eating periods were compared to noneating periods using a two-sample Kolmogorov–Smirnov test, with a significance level of 0.01 and a Bonferroni correction for the number of tested distributions (α = 2.2 × 10^−5^, Supplementary Table S1). All tests performed were two tailed.

## 3. Results

### 3.1. CNTY Electrodes Record Stable Spikes from Freely Moving Animals

**Figure 3:**
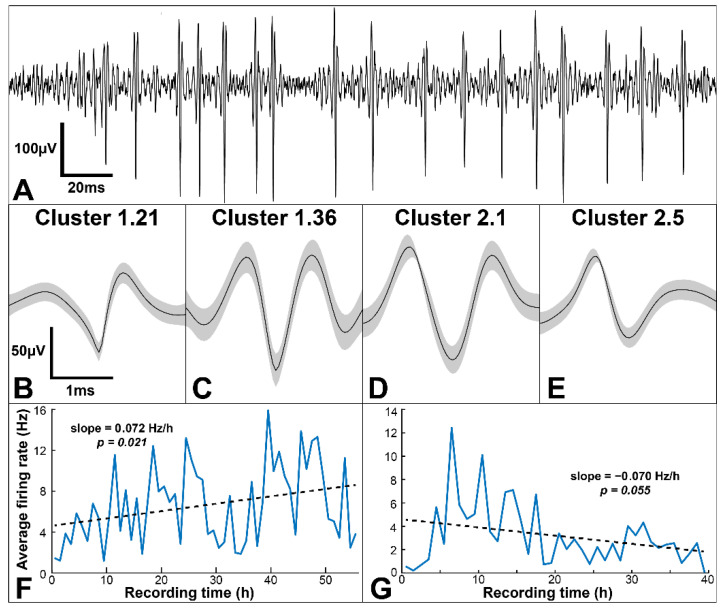
Spontaneous spikes recorded in freely moving animals. (**A**) Filtered ENG showing example recording spikes. (**B**–**E**) Example clusters sorted from recorded spikes in two animals. (**F**,**G**) Spike firing rate over recording time for two animals. Neither animal had a significant change in firing rate over time.

We have previously shown that CNTY electrodes can record spikes from the glossopharyngeal and vagus nerves in anesthetized rats and can be used to measure vagal tone in freely moving animals [26,27]. Here, we demonstrate a novel continuous chronic-recording setup (shown in Figure 1D,E) to record unanesthetized spiking activity which can be sorted into semi-distinct clusters. A total of four electrodes were implanted, two each in the left cervical vagus nerves of two rats, with an average impedance of 11.7 ± 6.5 kΩ at the time of implantation (measured at 1 kHz). Further measurements of CNTY electrode impedances for long-term implants have been published previously [26,27]. Figure 3A shows an example of filtered ENG with several recorded spikes, and Figure 3B–E show several example spike clusters from two animals. A total of 132 spike clusters were identified (56 in Rat 1, and 76 in Rat 2). Clusters are referred to as RatNumber.ClusterNumber (e.g., Cluster 1.21 is Cluster 21 from Rat 1). Average peak-to-peak amplitude of recorded spikes was 152 ± 97 µV for Rat 1 and 180 ± 162 µV for Rat 2. Spike SNR, defined as the average RMS of the spike waveforms compared to the RMS of the baseline, was 7.0 ± 4.9 for Rat 1, and 9.1 ± 5.3 for Rat 2. This is significantly larger than published SNR for acute recording with either the TIME or the LIFE electrodes [33,34]. Furthermore, median spike amplitude for all recorded spikes was stable over the recording time for Rat 2 and slightly increased over time for Rat 1, as shown in Supplementary Figure S1. Overall spike-firing rates were also consistent over the recording periods for both animals: Figure 3F,G show the average firing rates for each hour of recording, with least-squares regression lines showing no significant change in firing rate over time. Similarly, average spike SNR was stable over the recording time for both animals, as shown in Supplemental Figure S2. Thus, we are able to continuously record vagal spikes which have stable amplitude, SNR, and firing rates over time.

### 3.2. Spike Clusters’ Activity Is Correlated with Eating

Identifying the function of spontaneous spikes in freely moving animals is important to understanding how vagal fibers modulate their activity during normal animal behavior. Given the high ratio of gastric afferents in the vagus, most vagal spiking is involved with gastric signaling.

**Figure 4:**
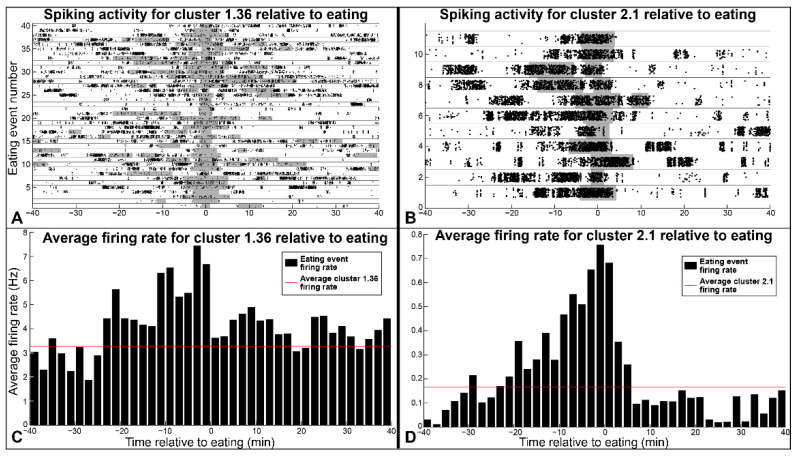
Example spiking activity related to eating. (**A**) Raster plot for Cluster 1.36. Grey-shaded boxes represent eating events, with dots representing spikes. (**B**) Raster plot for Cluster 2.1. (**C**) Firing rate of Cluster 1.36 relative to eating, averaged for all eating events. Red line represents the overall average firing rate of Cluster 1.36. (**D**) Firing rate of Cluster 2.1 relative to eating.

After animal-eating times were identified from video recordings, they were compared to the firing rates of individual spike clusters. In both animals, several clusters show a significant increase in firing rate that occurs <25 min before eating. Some clusters also had increased or decreased firing that occurred during eating, while others had increased firing that occurred <10 min after eating. Figure 4A,B show raster plots for one such spike cluster from each animal, with each row representing one eating event (shown by the shaded grey area). Figure 4C,D show the average firing rate of these clusters relative to the eating events, along with the overall average firing rate for each cluster. Cluster 1.36 (Figure 4A,C) had higher-than-average activity in the 25 min before eating, and higher-than-average activity in the 10 min following eating, with no change occurring during food consumption. Similarly, the firing rate of Cluster 2.1 is increased before and during eating, and unchanged after eating.

Many clusters exhibited a mix of behaviors, showing firing rates before, during, or after eating that were significantly different from baseline activity (*p* < 0.01 with Bonferroni correction). To analyze cluster behavior related to eating, clusters were sorted into groups based on their firing rate response before eating (from 25 min before, until the start of eating), during eating, and after eating (end of the eating event, until 10 min after eating). These data are summarized in Table 1 for both rats, which show how the cluster-firing rates changed for each group and the number of clusters from each animal which make up each group. The table shows the direction of change and associated p-value for the changes in firing rate of each group in the different eating-related periods (sum of the spiking activity in all clusters within a group compared to the baseline firing activity for the clusters in that group). Only 3 of the 132 recorded clusters did not showing any significant tuning to eating behavior. While specific spiking correlations are unique to each subject, they are consistent within each animal, and Figure 3 and Table 1 show that we can identify spike clusters that exhibit firing rate changes before, during, and after eating in both subjects.

### 3.3. Spike Cluster Interspike Intervals Show Changes in Bursting Related to Eating

**Figure 5:**
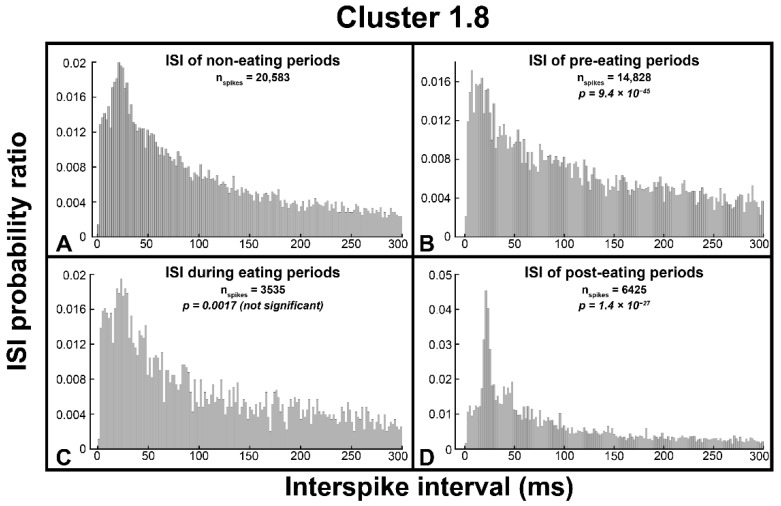
Interspike interval histograms for Cluster 1.8. (**A**) ISI histogram for noneating periods, which has a peak around 21 ms. (**B**) ISI histogram for pre-eating periods, which has a peak around 7ms, and a significantly different ISI distribution compared to noneating periods. (**C**) ISI histogram for eating periods, which has a peak around 23 ms and is not significantly different from noneating periods. (**D**) ISI histogram for post-eating periods, which has a peak around 21 ms and a secondary peak around 47 ms, and a significantly different ISI distribution compared to noneating periods.

Spikes are often observed exhibiting bursting behavior, where fibers tend to fire at specific frequencies. Bursting behavior can be seen in Figure 4A,B, where spikes appear in clumps. To quantify bursting, spike cluster interspike intervals (ISIs) were calculated for noneating, pre-eating, during eating, and posteating time windows. Eating-related distributions were compared to noneating distributions using a two-sample Kolmogorov–Smirnov test and were plotted in a histogram. Figure 5 shows ISI distributions for noneating, pre-eating, during eating, and posteating periods for one example cluster (Cluster 1.8, which is part of Group II and has increased activity before and after eating). In Figure 5A, we can see that the peak ISI of this cluster during noneating times is around 21 ms or a 48 Hz firing rate. However, in the 25 min before eating, this distribution shifts to the left, peaking instead at 7 ms or 143 Hz, signifying an increase in the bursting firing rate before eating. In the 10 min following eating, the bursting rate returns to the noneating value, though the ISI peak is more pronounced, meaning that bursting is a more prevalent spike behavior after eating. After eating, we also observe a secondary peak around 47 ms (21 Hz). During eating, the ISI distribution is not significantly different from noneating; thus, the bursting activity of Cluster 1.8 is changed before and after, but not during, eating behavior. In total, 10 clusters in Rat 1 and 18 clusters in Rat 2 demonstrated changes in ISI distribution related to eating.

These data are summarized in Supplemental Table S1, which shows *p*-values comparing noneating and eating-related ISI distributions for any cluster which showed a significant change. The 18 clusters in Rat 2 only showed a change in ISI distribution during eating, with no changes either before or after. The 10 clusters in Rat 1 each showed changes before eating, while some also had a significantly different ISI distribution during or after eating as well. Figure 5 and Supplementary Table S1 show that some of the spike clusters which are tuned to eating are observed to change bursting activity related to eating, though not all the clusters which show changes in overall activity have altered ISI/bursting behavior.

### 3.4. Spike-Cluster-Firing Rates Can Be Used to Classify Eating Compared to Other Behavior

**Figure 6:**
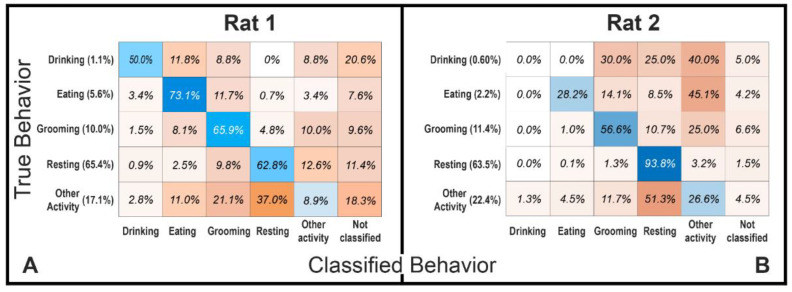
Confusion matrix for classifying animal behavior based on spike firing rates. Blue-colored cells show rates of correct classification, and orange-colored cells show rates of incorrect classification, such that each row sums to 100%. *Y*-axis labels show the percentage of recording time spent doing each behavior. (**A**) Confusion matrix for the classification of behavior in Rat 1. (**B**) Confusion matrix for the classification of behavior in Rat 2.

In addition to showing that individual spike clusters are correlated with food intake, we also examined whether spike-firing rates are sufficient to classify the times during which the animal is eating, compared to other behaviors, such as drinking, grooming, and resting. A multinomial logistic regression model was constructed, with behaviors and spike-cluster-firing rates averaged over 30 s. The model uses firing rates from each of the recorded clusters, as well as firing rates during peak delayed or preceding correlations with eating. The models were trained on the first 2/3 of recording data and tested on the final 1/3 of recording data. Figure 6 shows the confusion matrices for both animals, which show the performance of the model for classifying behavior with a probability threshold of π = 0.5 for classification. Percentages on the y-axis show the amount of time spent doing each behavior as a percentage of total recording time. In Rat 1, the model was able to classify eating most accurately, with a 73.1% accuracy. In Rat 2, the model performed best at classifying resting, with a 93.8% accuracy. Additionally, we can see by plotting the receiver operating characteristic (ROC) curves and the associated areas under the curve (AUC) in Supplementary Figure S3 that both models performed better than random chance for almost all behaviors (the only exception being classifying other activity in Rat 1). Overall, these results show that the firing rates of spontaneous vagal spikes sorted into clusters are sufficient to classify eating behavior in freely moving animals.

## 4. Discussion

CNTY electrodes are a promising neural interface: a small, low-impedance, and highly flexible biosensor ideal for interfacing with small peripheral nerves. Flexural rigidity, measured with an atomic-force microscope, shows that the CNTY electrodes are >10 times more flexible than PtIr electrodes of the same diameter (3.3 ± 1.5 × 10^−12^ N× m^2^ for the CNTY compared to 2.0 ± 0.57 × 10^−10^ N× m^2^ for the PtIr) [35]. Partly due to its small size (10 µm diameter) and flexibility, this axon-like biosensor has demonstrated stable, low impedance with chronic implantation, stable high SNR, and minimal evidence of chronic inflammation or nerve damage [26]. Furthermore, we have shown that CNTYs can be used for chronic recording in small autonomic nerves, such as the vagus and glossopharyngeal nerves, and for stimulation in larger somatic nerves and fascicles, such as the rat sciatic nerve [26]. Compared to previous intrafascicular interfaces, CNTYs provide higher SNR and improved stability and should be further investigated as a component of neural sensor devices.

Vagus-nerve-stimulation therapy is a rapidly growing field, with a wide variety of companies and studies investigating its use for treatment of a wide variety of diseases, including epilepsy, obesity, and heart failure [7,36,37,38]. However, many VNS studies have reported ambiguous results, pointing to the need for an improved understanding of natural vagal function, VNS mechanisms, and closed loop control of stimulation. Neural interfaces which allow for stable, high-SNR recordings are necessary for high-fidelity closed-loop control, and chronic recording in animal models may be used to better understand vagal function and response to therapy. In this study, we utilize the CNTY neural interface to show that eating-related spikes can be decoded from continuous chronic recordings in the vagus nerve, providing the first demonstration that spontaneous, physiologically specific signals can be recorded. We also show that spike-firing rate and interspike interval distributions show differing responses to physiological changes, which may be used as neural features for long-term recording and closed-loop systems, though further histological analysis may also be necessary to show the impact of chronic implantation on nerve health.

The vagus nerve contains afferent and efferent fibers that sense and control nearly every internal organ, playing a vital role in homeostasis, reflex pathways, and responses to physiological changes. While individual vagal-spiking activity has been recorded from isolated fibers and from acute intraneural recordings, to our knowledge, this is the first time spikes have been recorded in the vagus nerve in a chronic model. Combined with previous studies on recording average vagal RMS [27], we have demonstrated that various spontaneous and physiologically relevant signals can be recorded from the rat vagus nerve from freely moving animals using CNTY electrodes. Spike-firing rates stayed consistent for up to 56 recording hours (7–10 days after implantation), suggesting that the electrode interface is relatively stable during that time. Though spike clusters detected in the peripheral nervous system likely represent multiunit activity, Figure 2 shows that CNTY-recorded spikes can be sorted into clusters, allowing for more specialized decoding of relevant signals. Individual spike clusters recorded in freely moving rats show changes in firing rate before, during, and after eating; clusters were further sorted into different groups based on their firing behavior, as shown in Figure 3 and Table 1. Almost all recorded spike clusters showed increased activity up to 25 min before the start of eating. Firing rate also increased during and after eating for several cluster groups. Additionally, Figure 4 and Supplemental Table S1 show that some spike clusters exhibit changes in their ISI distribution during different eating phases. ISI distribution and peak ISI values are an important metric for describing spike-bursting behavior, and bursting frequencies may change independently of overall spiking activity in the cluster (due to clusters containing recordings from multiple individual axons). Thus, analysis of overall cluster activity and cluster ISI distributions are important metrics of vagal activity. Finally, Figure 5 and Supplementary Figure S3 shows that spike-cluster-firing rates can be used to accurately classify eating, drinking, grooming, and resting behavior, an important proof of concept for a closed-loop VNS system. Future studies could analyze how overall vagal-spiking activity and bursting rates respond to models of chronic disease, physiological stimuli (such as fasting), or to VNS treatment.

There are several important questions regarding the behavior of these fibers and how they might respond to physiological changes. One possibility is that these fibers may be related to the secretion or sensing of ghrelin and cholecystokinin (CCK) in the gut, peptide hormones that are known to regulate hunger and satiety via the vagus nerve and can even be found in small concentrations in the brain [30,39]. Ghrelin increases food intake and weight gain in rats and suppresses vagal activity of some gastric afferent fibers [30]. CCK, on the other hand, suppresses appetite while stimulating gastric afferent discharge [40,41]. However, the full picture relating the secretion of these hormones and their effects to vagus-nerve activity is not known. Future studies could investigate how administration of these peptides alters vagal activity in freely moving animals or how changes in vagal-spiking behavior correlate with changes in the concentration of gastric hormones in the gastrointestinal tract, in the blood, or in the CNS. Furthermore, changes in diet, such as high-fat or high-carbohydrate models of diet-induced obesity, have been shown to alter vagal satiety signaling and may have an effect on hunger signaling as well [14,42]. Utilizing the CNTY chronic-recording model described here would allow for insights into the effects of diet on vagal gastric signaling, and a more detailed measurement of food intake would allow for analysis of how differences in vagal spiking relate to differences in dietary behavior.

One important application for chronic recording of vagal activity is measuring the acute and chronic response of vagal signaling to various therapy approaches. High frequency VNS (vBLoc^®^) is thought to suppress afferent hunger signaling to reduce overeating in obese patients [43]. vBLoc^®^ stimulation is performed with a fixed on/off cycle; when stimulation is on, vagal activity is suppressed, which would decrease hunger signaling (e.g., the activity observed in cluster groups 1–5), and satiety signaling (e.g., cluster groups 1–3). However, studies have shown that vBLoc^®^ reduces hunger and *increases* satiety, though it is not clear how significant this effect is compared to a placebo [44]. More thorough investigation of the effects of vBLoc^®^ on hunger, satiety, and vagal activity are necessary to better understand therapy performance. The abiliti^®^ gastric stimulation system, on the other hand, is closed-loop, producing satiety during preprogrammed periods and in response to eating, thus reducing food intake and helping create a more stable meal schedule [45]. Patients with this system report improved self-control while eating, decreased binge eating, and reduced sensitivity to hunger [46], likely as a result of increased afferent activity during and after eating. Combining animal models of these stimulation systems with chronic spike recording using CNTY electrodes would allow for direct investigation of the mechanisms of these therapies [43]. Furthermore, chronic recording could be paired with other VNS paradigms, such as VNS for epilepsy or depression, to investigate possible off-target side effects.

Overall, our results show that it is possible to record chronic signals in the rat vagus nerve continuously, opening the door for studies that were previously not possible. Furthermore, the ability to detect and decode spontaneous spiking activity from chronic vagal recordings could allow for a more detailed analysis of vagus-nerve response to changes in diet, therapy, and behavior. This technology could be used to develop closed-loop VNS for metabolic disorders, which adapt stimulation based on recorded vagal activity. Chronic recording in animal models can also be used to further study the vagal pathways that control food intake and how they respond to VNS and other treatments.

## Figures and Tables

**Table 1:** Firing rates of cluster groups relative to eating. Sorted clusters are separated into five cluster groups based on their response to eating. Table shows the number of clusters of each group recorded in both animals and the behavior of those cluster groups before, during, and after eating: up arrow/green color means an increased firing rate, dash/yellow color means no change in firing rate, and down arrow/red color means a decreased firing rate for the cluster group.

Cluster Group	Rat 1	Rat 2	Before Eating		During Eating		After Eating	
Group I	19	0	↑	*p* << 0.0001	↑	*p* << 0.0001	↑	*p* << 0.0001
Group II	13	13	↑	*p* << 0.0001	─	*p* = 0.024	↑	*p* << 0.0001
Group III	24	0	↑	*p* << 0.0001	↓	*p* ≤ 0.001	↑	*p* << 0.0001
Group IV	0	59	↑	*p* << 0.0001	↑	*p* = << 0.0001	─	*p* = 0.95
Group V	0	1	↑	*p* << 0.0001	─	*p* = 0.0093	↓	*p* = << 0.0001

## Data Availability

The data that support the findings of this study are available from the corresponding author upon reasonable request. Some custom-analysis code was created to analyze spiking activity and is available from the corresponding author upon reasonable request.

## Supplementary Materials

The following supporting information can be downloaded at: https://www.mdpi.com/article/10.3390/bios12020114/s1, Supplementary Figure S1. Median spike amplitude over time. (A). Median spike amplitude for spikes recorded in Rat 1 slightly increase over the recording time, with a statistically significant slope of 0.39µV per hour. (B). Median spike amplitude of spikes recorded in Rat 2 did not change over the recording time. Supplementary Figure S2. Average spike SNR (spike RMS divided by noise RMS) over time. (A). SNR of spikes recorded in Rat 1 did not change over the recording time. (B). SNR of spikes recorded in Rat 2 did not change over the recording time. Supplementary Table S1. Differences in ISI distributions for before, during, and after eating periods, compared to non-eating periods, for all clusters which had at least one group with a significant change. Cluster groups are shown for each cluster (see Table 1), and non-significant p-values are not shown. Supplementary Figure S3. Receiver operating characteristic (ROC) curves and area-under-the-curve (AUC) values to assess performance of a multinomial logistic regression model to classify animal behaviors based on spike cluster firing rates. Dotted lines show the expected ROC curve for a random classifier. (A). ROC curve for classifying drinking in Rat 1, with AUC = 0.86. (B). ROC curve for classifying drinking in Rat 2, with AUC = 0.62. (C). ROC curve for classifying eating in Rat 1, with AUC = 0.94. (D). ROC curve for classifying eating in Rat 2, with AUC = 0.82. (E). ROC curve for classifying grooming in Rat 1, with AUC = 0.88. (F). ROC curve for classifying grooming in Rat 2, with AUC = 0.90. (G). ROC curve for classifying resting in Rat 1, with AUC = 0.82. (H). ROC curve for classifying resting in Rat 2, with AUC = 0.86. I: ROC curve for classifying other activity in Rat 1, with AUC = 0.47. (J). ROC curve for classifying other activity in Rat 2.
