# Supplementary material for: Decoding Vagus-Nerve Activity with Carbon Nanotube Sensors in Freely Moving Rodents
Source: Biosensors (Basel). 2022 Feb 11;12(2):114. doi: 10.3390/bios12020114 (PMC8870245; doi:10.3390/bios12020114)
Supplement: Supplementary file 1 [file biosensors-12-00114-s001.zip › biosensors-1569057-supplementary.pdf]

biosensors

Supplementary Information

Title

Decoding Vagus-Nerve Activity with Carbon Nanotube Sensors in Freely Moving Rodents

Joseph T. Marmerstein, Grant A. McCallum and Dominique M. Durand *

Neural Engineering Center, Biomedical Engineering, Case Western Reserve University, Cleveland, OH 44106, USA; jtm124@case.edu (J.T.M.); gam19@case.edu (G.A.M.)
* Correspondence: dxd6@case.edu

Citation: Marmerstein, J.T.; McCallum, G.A.; Durand, D.M. Decoding Vagus-Nerve Activity with Carbon Nanotube Sensors in Freely Moving Rodents. *Biosensors* **2022**, *12*, 114. https://doi.org/10.3390/bios12020114

Received: 8 January 2022
Accepted: 5 February 2022
Published: 11 February 2022

Publisher's Note: MDPI stays neutral with regard to jurisdictional claims in published maps and institutional affiliations.

Copyright: © 2022 by the authors. Submitted for possible open access publication under the terms and conditions of the Creative Commons Attribution (CC BY) license (https://creativecommons.org/licenses/by/4.0/).

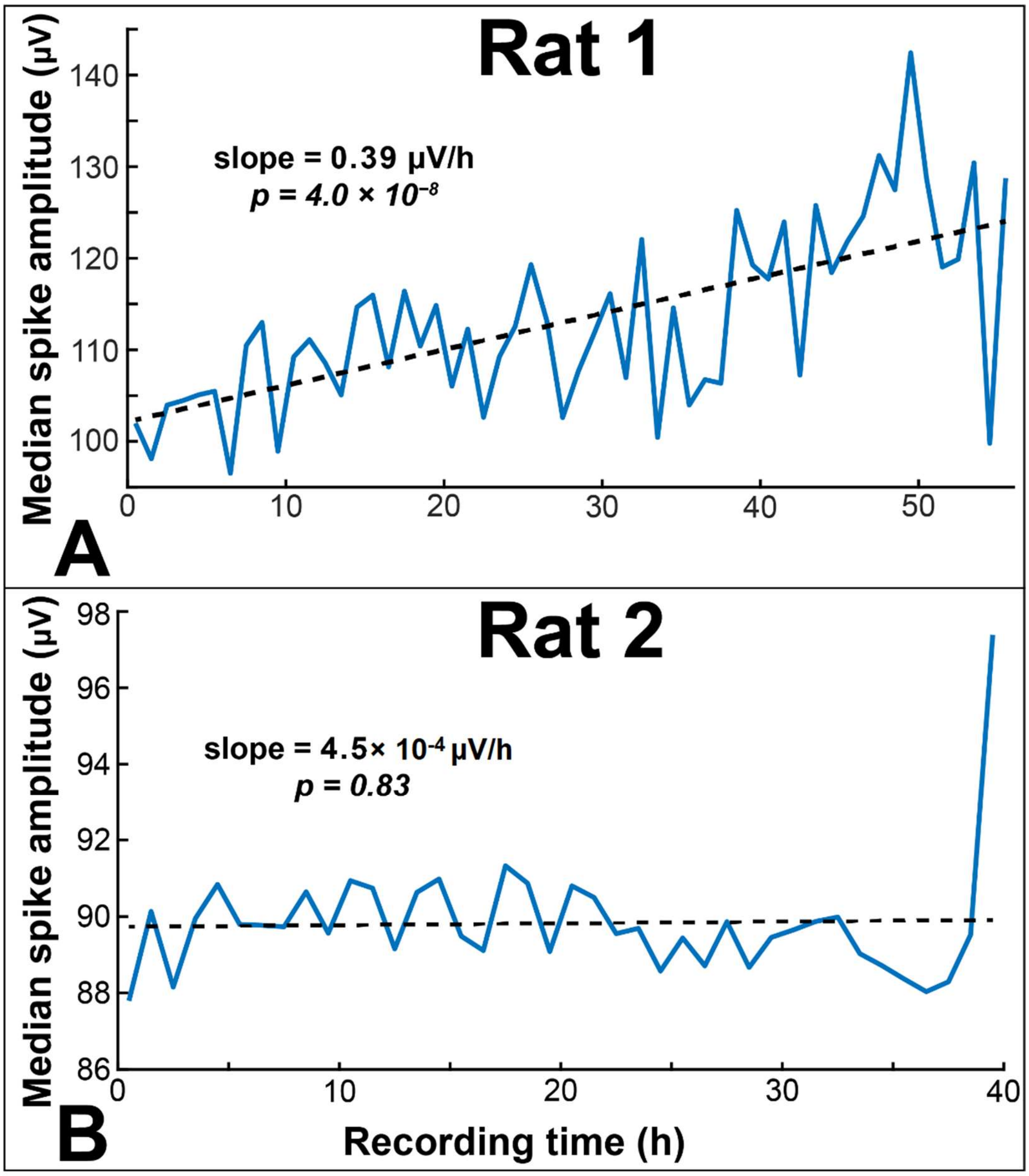

Supplementary Figure S1. Median spike amplitude over time. × 10^{-4} μV/h (A). Median spike amplitude for spikes recorded in Rat 1 slightly increase over the recording time, with a statistically

significant slope of 0.39μV per hour. (**B**). Median spike amplitude of spikes recorded in Rat 2 did not change over the recording time.

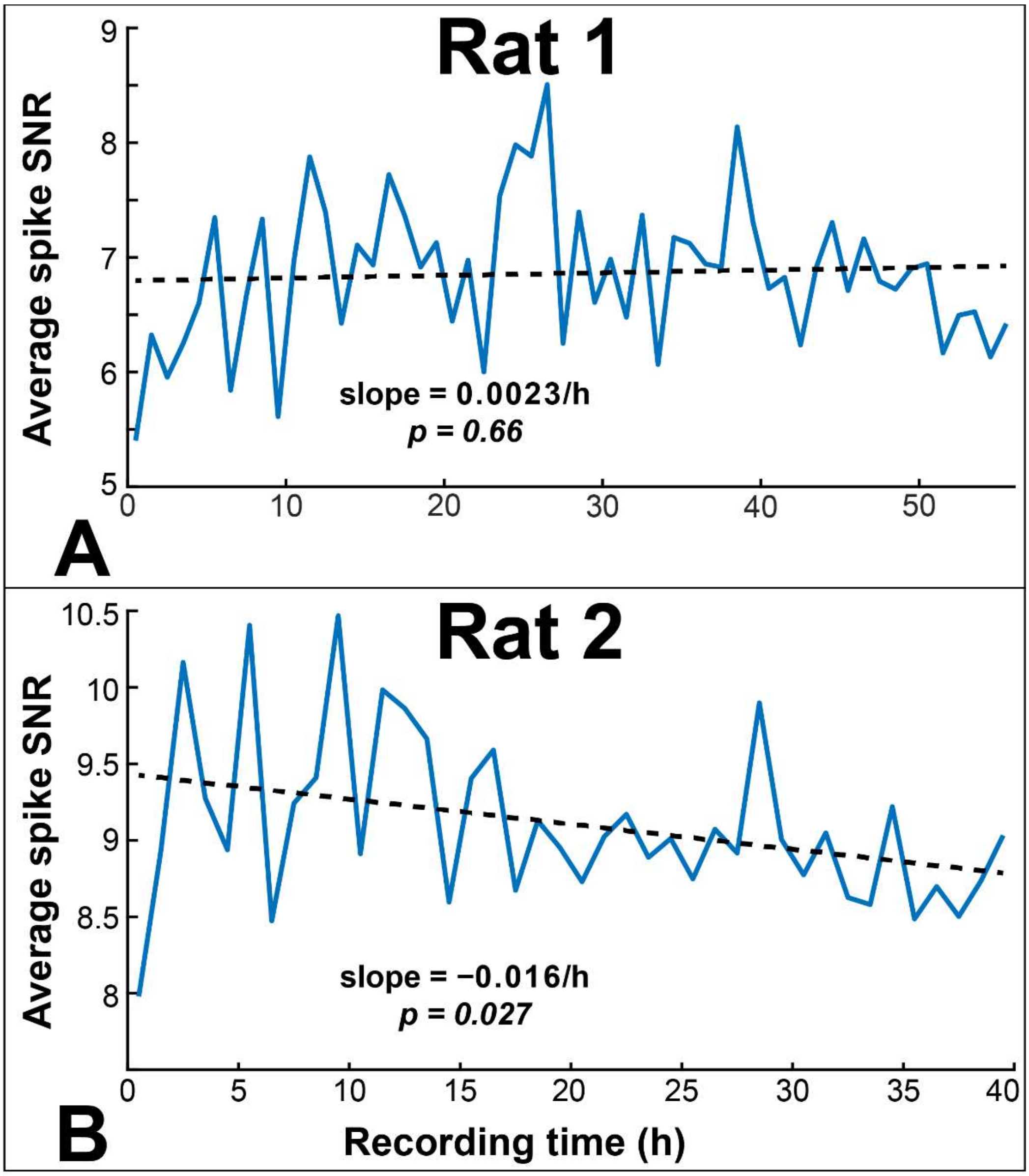

Supplementary Figure S2. Average spike SNR (spike RMS divided by noise RMS) over time. **(A).** SNR of spikes recorded in Rat 1 did not change over the recording time. **(B).** SNR of spikes recorded in Rat 2 did not change over the recording time.

Supplementary Table S1. Differences in ISI distributions for before, during, and after eating periods, compared to non-eating periods, for all clusters which had at least one group with a significant change. Cluster groups are shown for each cluster (see Table 1), and non-significant p-values are not shown.

Cluster Number	Cluster Group	Before Eating	During Eating	After Eating
1.8	II	9.4×10^{-45}	NS	1.4×10^{-27}
1.14	II	3.2×10^{-10}	2.1×10^{-5}	7.3×10^{-7}
1.18	II	6.9×10^{-13}	NS	NS
1.20	II	5.7×10^{-11}	NS	NS
1.21	II	9.9×10^{-11}	NS	NS

1.28	II	1.9×10^{-9}	NS	NS
1.29	II	4.4×10^{-9}	NS	NS
1.30	IV	2.5×10^{-6}	NS	NS
1.52	IV	4.0×10^{-10}	NS	NS
1.56	IV	6.4×10^{-13}	8.2×10^{-9}	1.4×10^{-11}
2.10	IV	NS	3.6×10^{-11}	NS
2.11	IV	NS	4.9×10^{-8}	NS
2.12	IV	NS	2.4×10^{-8}	NS
2.13	IV	NS	5.1×10^{-7}	NS
2.16	IV	NS	6.3×10^{-6}	NS
2.18	IV	NS	9.6×10^{-10}	NS
2.21	IV	NS	8.8×10^{-9}	NS
2.24	IV	NS	3.9×10^{-6}	NS
2.30	IV	NS	3.1×10^{-6}	NS
2.31	IV	NS	1.0×10^{-5}	NS
2.33	IV	NS	7.7×10^{-12}	NS
2.34	IV	NS	5.5×10^{-7}	NS
2.37	IV	NS	2.3×10^{-10}	NS
2.40	IV	NS	2.8×10^{-10}	NS
2.42	IV	NS	2.0×10^{-5}	NS
2.43	IV	NS	8.2×10^{-6}	NS
2.58	IV	NS	1.7×10^{-5}	NS
2.73	IV	NS	2.6×10^{-6}	NS

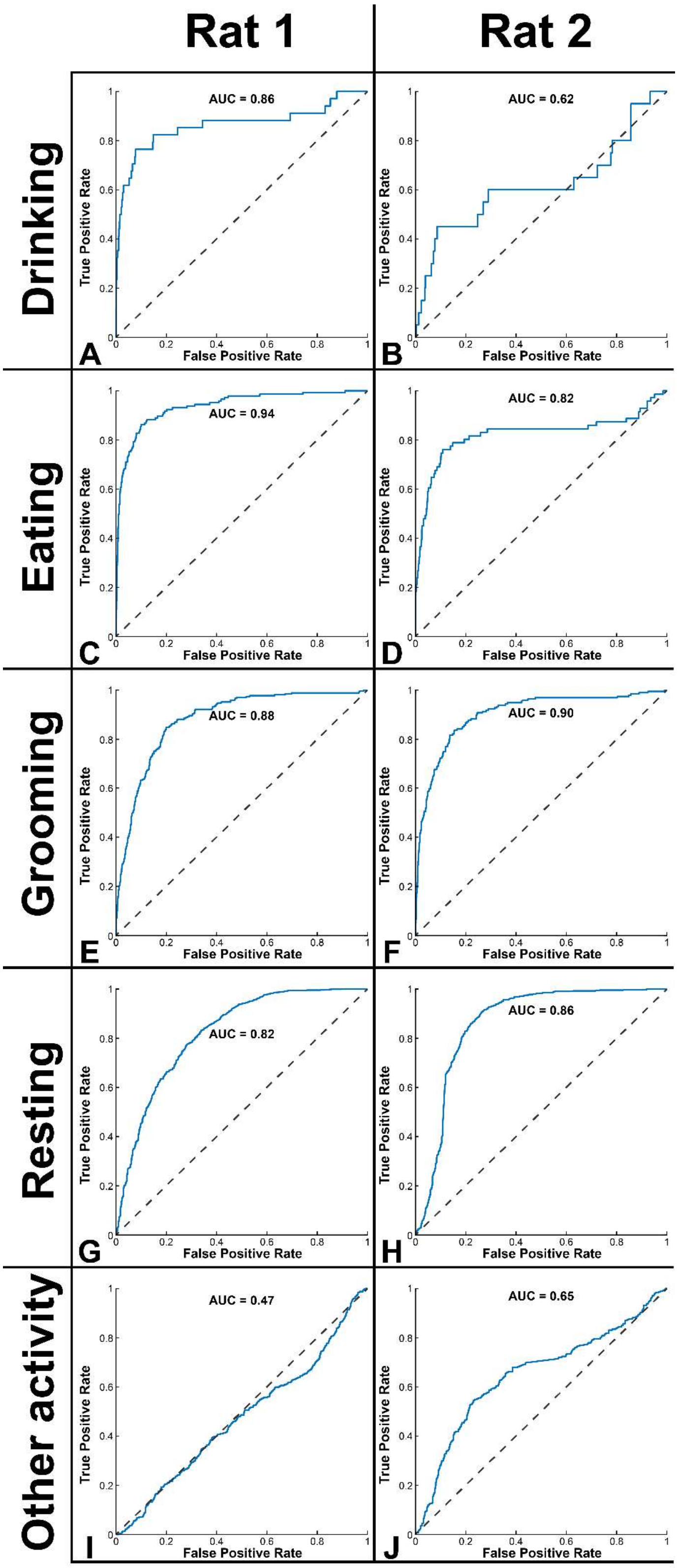

Supplementary Figure S3. Receiver operating characteristic (ROC) curves and area-under-the-curve (AUC) values to assess performance of a multinomial logistic regression model to classify animal behaviors based on spike cluster firing rates. Dotted lines show the expected ROC curve for a random classifier. **(A).** ROC curve for classifying drinking in Rat 1, with AUC = 0.86. **(B).** ROC curve for classifying drinking in Rat 2, with AUC = 0.62. **(C).** ROC curve for classifying eating in Rat 1, with AUC = 0.94. **(D).**

ROC curve for classifying eating in Rat 2, with AUC = 0.82. **(E).** ROC curve for classifying grooming in Rat 1, with AUC = 0.88. **(F).** ROC curve for classifying grooming in Rat 2, with AUC = 0.90. **(G).** ROC curve for classifying resting in Rat 1, with AUC = 0.82. **(H).** ROC curve for classifying resting in Rat 2, with AUC = 0.86. **I:** ROC curve for classifying other activity in Rat 1, with AUC = 0.47. **(J).** ROC curve for classifying other activity in Rat 2.
